# Supplementary material for: Pathological pallidal beta activity in Parkinson’s disease is sustained during sleep and associated with sleep disturbance
Source: Nat Commun. 2023 Sep 5;14:5434. doi: 10.1038/s41467-023-41128-6 (PMC10480217; doi:10.1038/s41467-023-41128-6)
Supplement: Supplementary file 1 — Supplementary Information [file 41467_2023_41128_MOESM1_ESM.pdf]

Supplementary information

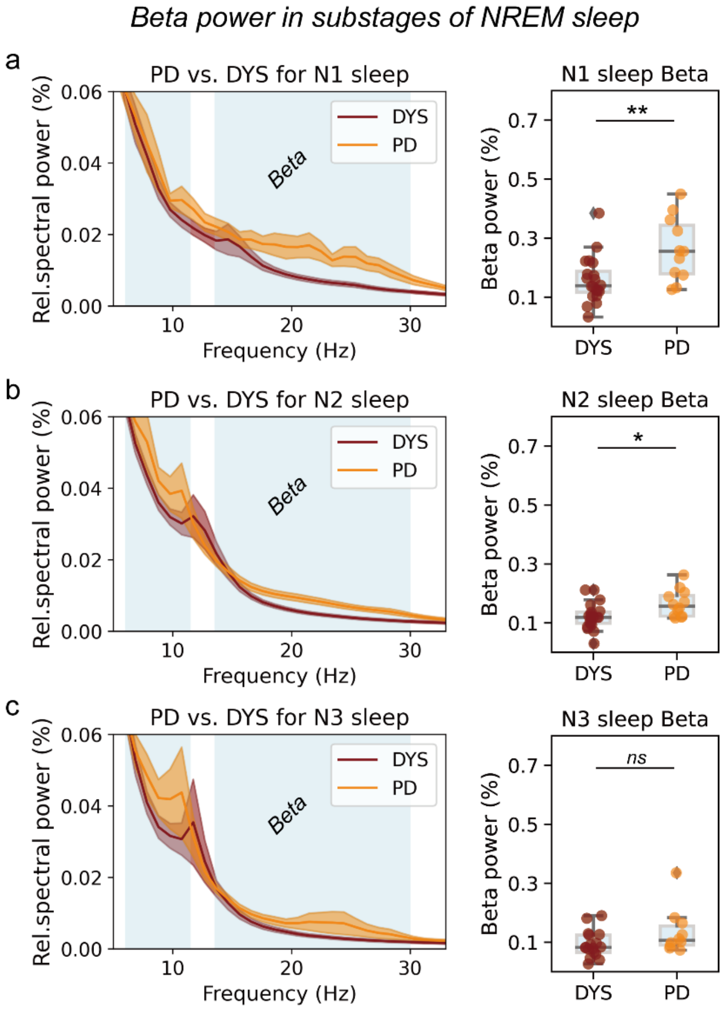

Supplementary Fig. 1 Beta power in substages of non-rapid eye movement (NREM) sleep in Parkinson's disease and dystonia. a, b, and **c** demonstrate the comparisons of power spectra (left) and beta power (right) between Parkinson's disease and dystonia in N1, N2, and N3 sleep, respectively. Beta power is significantly higher in Parkinson's disease than dystonia in N1 (** $P = 0.006$, n for Parkinson's disease subjects = 11; n for dystonia subjects = 20, two-sided Mann–Whitney U test) and N2 (* $P = 0.013$, n for Parkinson's disease subjects = 12; n for dystonia subjects = 20, two-sided Mann–Whitney U test), but not N3 sleep ($P = 0.093$, n for Parkinson's disease subjects = 10; n for dystonia subjects = 17, two-sided Mann–Whitney U test). Shaded areas in all spectrum plots represent SEM. For all box plots, the lower and upper borders of the box represent the 25th and 75th percentiles, respectively. The centerline represents the median. The whiskers extend to the smallest and largest data points that are not outliers (1.5 times the interquartile range). Source data are provided as a Source Data file.

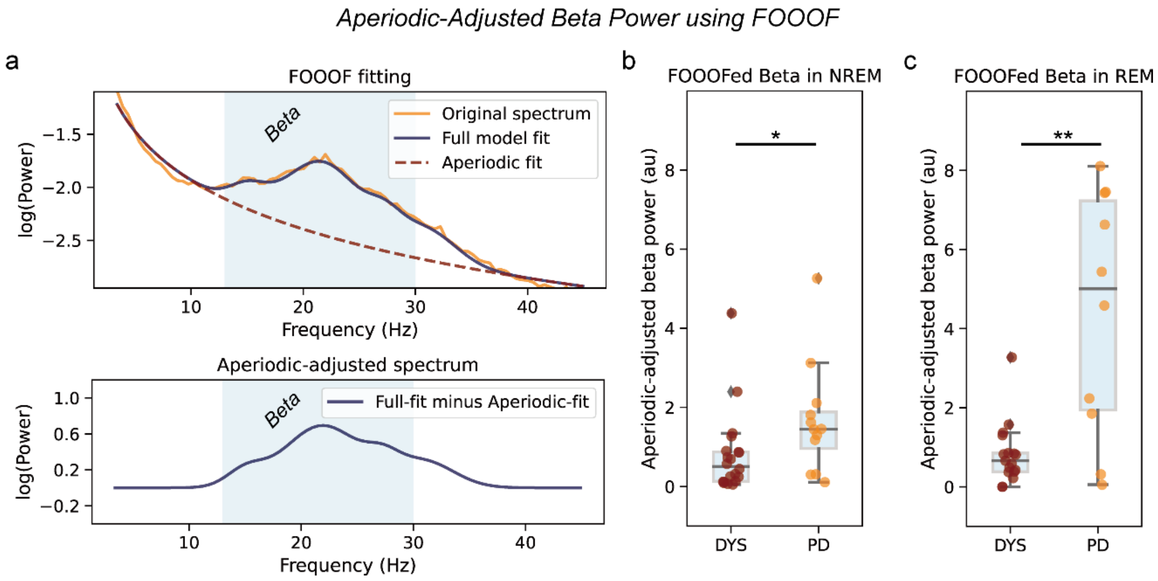

Supplementary Fig. 2 Periodic beta activity after spectral parameterization in non-rapid eye movement (NREM) and REM sleep in Parkinson's disease and dystonia. a demonstrates the process of spectral parameterization by applying fitting oscillations & one over f (FOOOF) algorithm on REM sleep data from subject PD-9. The upper plot shows the original spectrum, full model fitted spectrum, and the aperiodic fitted spectrum. The beta band range is highlighted in blue. The bottom plot shows the aperiodic-adjusted spectrum which is obtained by subtracting the aperiodic fitted spectrum from the full model fitted spectrum. **b** shows the comparison of aperiodic-adjusted beta power (a.u.) in NREM sleep between subjects with dystonia and Parkinson's disease (* $P = 0.019$, n for Parkinson's disease subjects = 12; n for dystonia subjects = 20, two-sided Mann–Whitney U test). **c** shows the comparison of aperiodic-adjusted beta power (a.u., arbitrary units) in REM sleep between subjects with dystonia and Parkinson's disease (** $P = 0.007$, n for Parkinson's disease subjects = 12; n for dystonia subjects = 20, two-sided Mann–Whitney U test). For all box plots, the lower and upper borders of the box represent the 25th and 75th percentiles, respectively. The centerline represents the median. The whiskers extend to the smallest and largest data points that are not outliers (1.5 times the interquartile range). Source data are provided as a Source Data file.

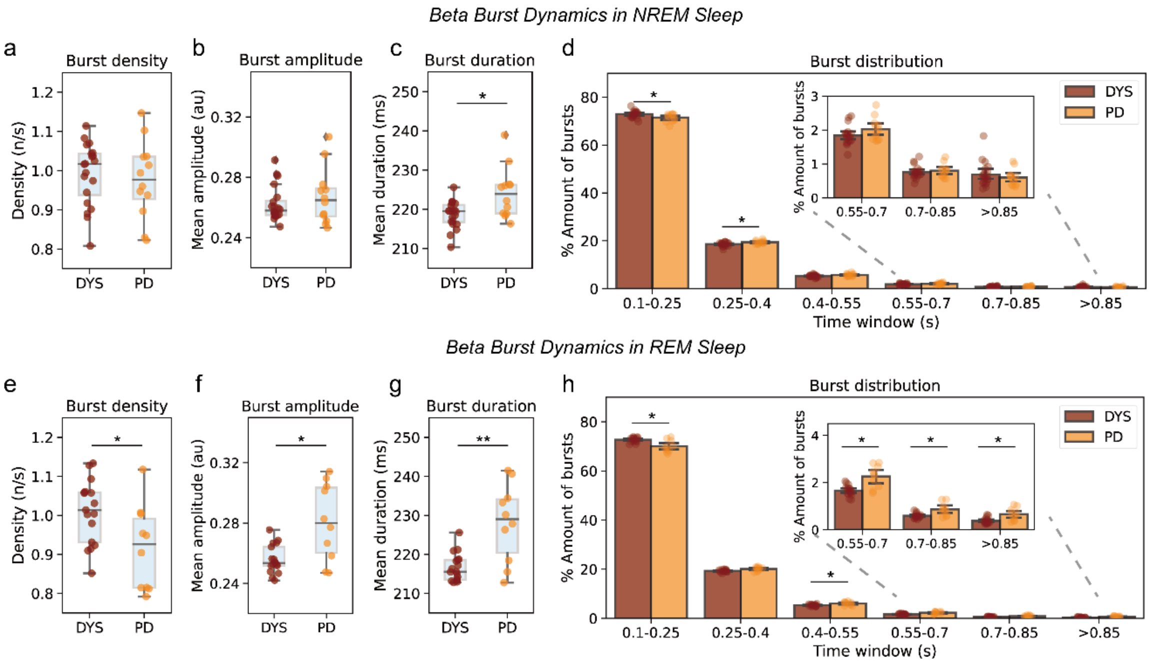

Supplementary Fig. 3 The comparisons of beta burst dynamic in non-rapid eye movement (NREM) and REM sleep between Parkinson's disease and dystonia. a, **b**, and **c** show the average density (*P* = 0.521, Mann–Whitney *U* test), amplitude (*P* = 0.683, Mann–Whitney *U* test), and duration (* *P* = 0.013, Mann–Whitney *U* test) of beta burst in NREM sleep in dystonia and Parkinson's disease. **d** shows the burst duration distribution in dystonia and Parkinson's disease ($P_{Bonferroni}$ = 0.024, 0.036, 0.162, 0.744, 0.999, and 0.999 for burst duration of 0.1-0.25, 0.25-0.4, 0.4-0.55, 0.55-0.7, 0.7-0.85, and >0.85 seconds, respectively, Mann–Whitney *U* test). **e**, **f**, and **g** show the average density (* *P* = 0.047, Mann–Whitney *U* test), amplitude (* *P* = 0.020, Mann–Whitney *U* test), and duration (** *P* = 0.005, Mann–Whitney *U* test) of beta burst in REM sleep in dystonia and Parkinson's disease. **h** shows the burst duration distribution in dystonia and Parkinson's disease in REM sleep ($P_{Bonferroni}$ = 0.048, 0.077, 0.042, 0.012, 0.036, and 0.018 for burst duration of 0.1-0.25, 0.25-0.4, 0.4-0.55, 0.55-0.7, 0.7-0.85, and >0.85 seconds, respectively, Mann–Whitney *U* test). All tests were done with the null hypothesis defined as two-sided. For the comparisons in NREM sleep, *n* for Parkinson's disease subjects = 12; *n* for dystonia subjects = 20. For the comparisons in REM sleep, *n* for Parkinson's disease subjects = 10; *n* for dystonia subjects = 17. For all box plots, the lower and upper borders of the box represent the 25th and 75th percentiles, respectively. The centerline represents the median. The whiskers extend to the smallest and largest data points that are not outliers (1.5 times the interquartile range). All bar plots indicate mean ± SEM. Source data are provided as a Source Data file.

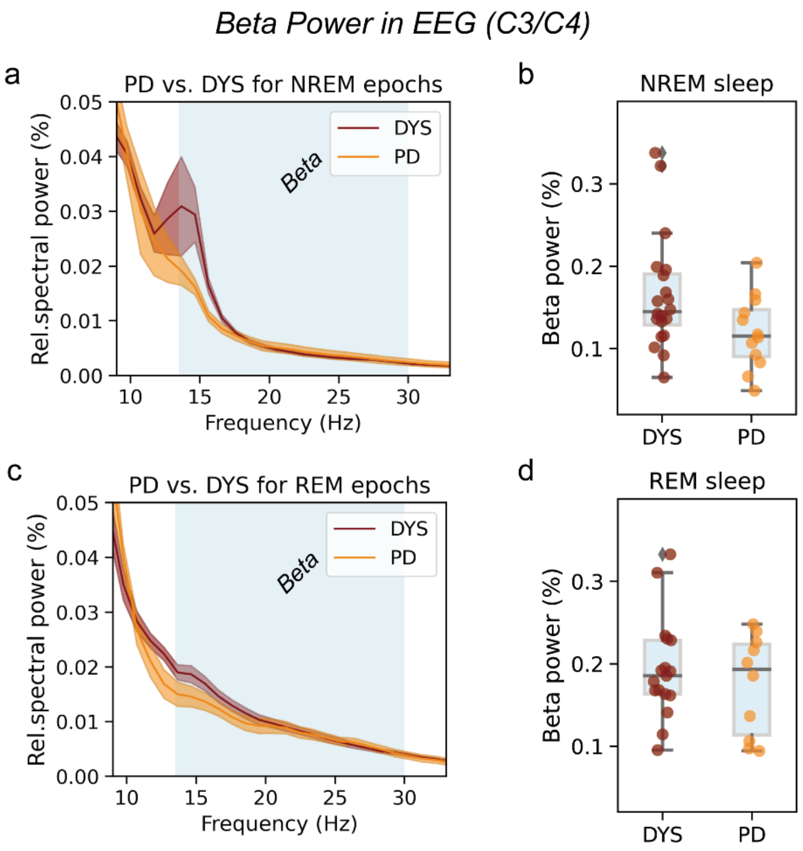

Supplementary Fig. 4 Beta power in electroencephalogram (EEG) in non-rapid eye movement (NREM) and REM sleep between Parkinson's disease and dystonia. a shows the comparison of the power spectra in NREM sleep between dystonia and Parkinson's disease in EEG. EEG data are obtained as the average of the C3 and C4 channels. Note that the spectral peak occurred at around 13 Hz in dystonia subjects could be caused by sleep spindles. **b** shows the average beta band power in EEG in NREM sleep in subjects with dystonia and Parkinson's disease (P = 0.070, n for Parkinson's disease subjects = 12; n for dystonia subjects = 20, two-sided Mann–Whitney U test). **c** shows the comparison of the power spectra in REM sleep between dystonia and Parkinson's disease in EEG. **d** shows the average beta band power in EEG in REM sleep in dystonia and Parkinson's disease (P = 0.782, n for Parkinson's disease subjects = 10; n for dystonia subjects = 17, two-sided Mann–Whitney U test). Shaded areas in all spectrum plots represent SEM. For all box plots, the lower and upper borders of the box represent the 25th and 75th percentiles, respectively. The centerline represents the median. The whiskers extend to the smallest and largest data points that are not outliers (1.5 times the interquartile range). Source data are provided as a Source Data file.

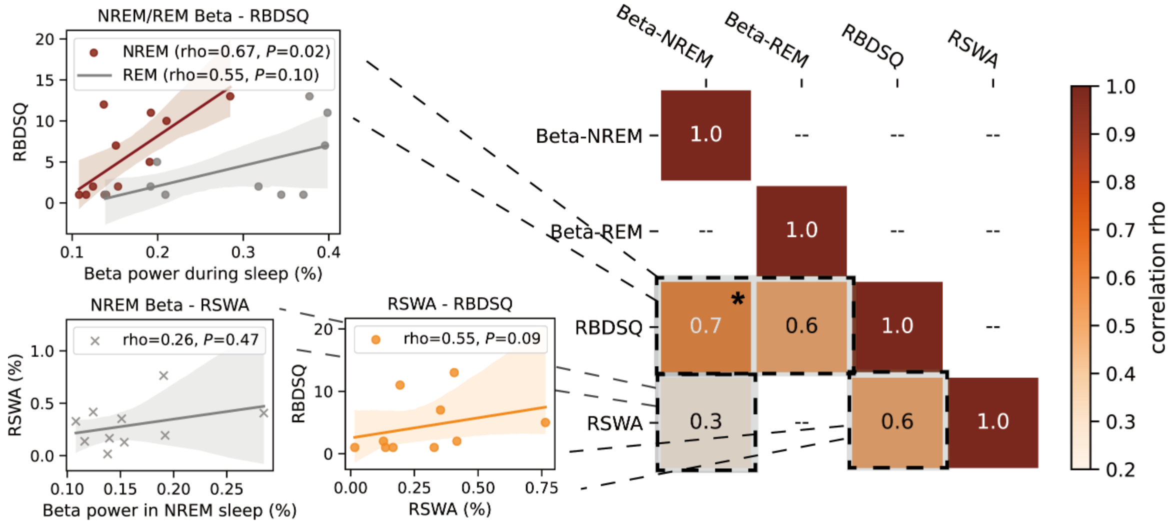

Supplementary Fig. 5 Correlations between pallidal beta band power and REM sleep behavior disorder severity ratings. The heatmap on the right shows that beta power in NREM (Spearman *rho* = 0.67, * *P* = 0.02), but not REM sleep is significantly correlated with the REM sleep behavior disorder-screening questionnaire (RBDSQ) score. However, no correlation is observed between beta power in NREM sleep and the time proportion/severity of REM sleep without atonia (RSWA) (Spearman *rho* = 0.26, *P* = 0.47), despite that the severity of RSWA is moderately correlated with the RBDSQ score (Spearman *rho* = 0.55, *P* = 0.09). Scatter plots for the abovementioned correlations are shown separately on the left. The error bands in all scatter plots are the 95% confidence interval for the regression estimate. All tests were done with the null hypothesis defined as two-sided. Source data are provided as a Source Data file.
