# Supplementary material for: Pathological pallidal beta activity in Parkinson’s disease is sustained during sleep and associated with sleep disturbance
Source: Nat Commun. 2023 Sep 5;14:5434. doi: 10.1038/s41467-023-41128-6 (PMC10480217; doi:10.1038/s41467-023-41128-6)
Supplement: Supplementary file 3 — Reporting Summary [file 41467_2023_41128_MOESM3_ESM.pdf]

nature portfolio

Corresponding author(s): Jianguo Zhang and Wolf-Julian Neumann

Last updated by author(s): Aug 8, 2023

Reporting Summary

Nature Portfolio wishes to improve the reproducibility of the work that we publish. This form provides structure for consistency and transparency in reporting. For further information on Nature Portfolio policies, see our Editorial Policies and the Editorial Policy Checklist.

Statistics

For all statistical analyses, confirm that the following items are present in the figure legend, table legend, main text, or Methods section.

n/a	Confirmed	
☐	☒	The exact sample size (*n*) for each experimental group/condition, given as a discrete number and unit of measurement
☐	☒	A statement on whether measurements were taken from distinct samples or whether the same sample was measured repeatedly
☐	☒	The statistical test(s) used AND whether they are one- or two-sided<br>*Only common tests should be described solely by name; describe more complex techniques in the Methods section.*
☐	☒	A description of all covariates tested
☐	☒	A description of any assumptions or corrections, such as tests of normality and adjustment for multiple comparisons
☐	☒	A full description of the statistical parameters including central tendency (e.g. means) or other basic estimates (e.g. regression coefficient) AND variation (e.g. standard deviation) or associated estimates of uncertainty (e.g. confidence intervals)
☐	☒	For null hypothesis testing, the test statistic (e.g. *F*, *t*, *r*) with confidence intervals, effect sizes, degrees of freedom and *P* value noted<br>*Give P values as exact values whenever suitable.*
☒	☐	For Bayesian analysis, information on the choice of priors and Markov chain Monte Carlo settings
☐	☒	For hierarchical and complex designs, identification of the appropriate level for tests and full reporting of outcomes
☐	☒	Estimates of effect sizes (e.g. Cohen's *d*, Pearson's *r*), indicating how they were calculated

Our web collection on statistics for biologists contains articles on many of the points above.

Software and code

Policy information about availability of computer code

Data collection	We used the JE-212 amplifier (Nihon Kohden, Tokyo, Japan) to record PSG and LFP data.
Data analysis	We used Python 3.8 (packages including MNE-Python [version 1.2.3], SciPy [version 1.7.3] and NumPy [version 1.20.2]) for the analysis of neurophysiological data.<br>We used MATLAB 2019b (LEAD-DBS [version 2.5.3]) to process all neuroimaging data.<br>All relevant codes employed in the study can be freely accessed at https://github.com/zixiao-yin/SleepBeta (Zenodo https://doi.org/ 10.5281/zenodo.818078058[1].)<br>[1] Yin, Z. et al. Beta during sleep in PD Code/Data: v1.0725.2023. (2023) doi:10.6084/M9.FIGSHARE.23740410.

For manuscripts utilizing custom algorithms or software that are central to the research but not yet described in published literature, software must be made available to editors and reviewers. We strongly encourage code deposition in a community repository (e.g. GitHub). See the Nature Portfolio guidelines for submitting code & software for further information.

Data

Policy information about availability of data

All manuscripts must include a data availability statement. This statement should provide the following information, where applicable:

- Accession codes, unique identifiers, or web links for publicly available datasets
- A description of any restrictions on data availability
- For clinical datasets or third party data, please ensure that the statement adheres to our policy

Due to the data protection regulations of Beijing Tiantan Hospital and Medical Capital University, the raw sleep electrophysiological data used in this study are available from the corresponding author (JGZ) after approval of the IRB of Beijing Tiantan Hospital (E-mail: ttyyirb@163.com, Tel: +86 10 5997 8555). We are happy to share our data and provide assistance in obtaining the approval upon request. It typically takes 2-4 weeks to get the approval, depending on the amount of data required, how the data will be used, etc. Source data are provided with this paper.

Human research participants

Policy information about studies involving human research participants and Sex and Gender in Research.

Reporting on sex and gender	This study included 20 patients with dystonia (10 female patients) and 12 patients with Parkinson's disease (6 female patients). Consent has been obtained for reporting individual-level but de-identified data. We did not conduct sex- and gender-based analyses as the aim of the study is to investigate the role of basal ganglia beta oscillations in sleep disorders in Parkinson's disease. There is no sufficient evidence showing that sex or gender would have an impact on quantifying this role. But in the source data file, the data are shown disaggregated by sex.
Population characteristics	Twelve subjects with Parkinson's disease (PD) and twenty subjects with dystonia undergoing DBS electrode implantation in the internal pallidum were recruited for electrophysiological recordings during sleep. The median (IQR) age is 59.0 (15.0) for the PD group and 54.0 (18.0) for the dystonia group.
Recruitment	Thirty-two subjects with movement disorders (twelve with PD, twenty with dystonia) scheduled to receive GPi-DBS implantation at Beijing Tiantan Hospital were included. The inclusion criteria were: (i) for primary dystonia patients, predominantly cervical or oromandibular dystonia without prominent limb involvement; (ii) for PD patients, unquestioned diagnosis of PD based on the UK brain bank criteria, and (iii) for all patients, the capacity to cooperate with whole-night polysomnography recordings and the absence of cerebral lesions on magnetic resonance imaging (MRI) such as tumor and stroke. Standard motor assessments using Toronto Western Spasmodic Torticollis Rating/ Burke–Fahn–Marsden Dystonia Rating Scale (for dystonia), or MDS-UPDRS (for PD, on and off dopaminergic medication) were conducted before the surgery. Subjective sleep quality was evaluated using the PSQI for all subjects and RBD was assessed using the RBD severity questionnaire for PD subjects.<br>Patients were asked whether they are willing to participate in the study during lead externalization. Patients who agreed to participate may have higher degrees of sleep disturbances because those who have good sleep qualities may consider sleep monitoring unnecessary. However, we do not think this will significantly impact our results as there are patients who sleep well did agree to participate (e.g., #PD-11 and #Dyst-6). The patients did not receive payment for their participation.
Ethics oversight	This study is in agreement with the Declaration of Helsinki, and is approved by the IRB of Beijing Tiantan Hospital (HX-A-2021006). All subjects provided written informed consent.

Note that full information on the approval of the study protocol must also be provided in the manuscript.

Field-specific reporting

Please select the one below that is the best fit for your research. If you are not sure, read the appropriate sections before making your selection.

[x] Life sciences [ ] Behavioural & social sciences [ ] Ecological, evolutionary & environmental sciences

For a reference copy of the document with all sections, see nature.com/documents/nr-reporting-summary-flat.pdf

Life sciences study design

All studies must disclose on these points even when the disclosure is negative.

Sample size	Overall, 32 patients were included in this study. We employed no statistical models to predetermine sample size, but our sample sizes are at the same level as or larger than those reported in previous studies with similar research aims. For example, in 2018 JNNP paper [1], 10 PD patients were recruited for simultaneously PSG-LFP recordings during sleep; in 2018 TNSRE paper [2], 12 PD patients were recorded during sleep; and in 2022 NPJ Parkinsons Dis paper [3], 4 PD patients were recorded during sleep.<br>[1] Thompson J A, Tekriwal A, Felsen G, Ozturk M, Telkes I, Wu J, Ince N F, Abosch A. Sleep patterns in Parkinson's disease: direct recordings from the subthalamic nucleus[J]. Journal of Neurology, Neurosurgery & Psychiatry, 2018, 89(1): 95–104.<br>[2] Chen Y, Gong C, Hao H, Guo Y, Xu S, Zhang Y, Yin G, Cao X, Yang A, Meng F, Ye J, Liu H, Zhang J, Sui Y, Li L. Automatic Sleep Stage Classification Based on Subthalamic Local Field Potentials[J]. IEEE Trans Neural Syst Rehabil Eng, 2019, 27(2): 118–128.

[3] Verma A K, Acosta Lenis S F, Aman J E, Sanabria D E, Wang J, Pearson A, Hill M, Patriat R, Schrock L E, Cooper S E, Park M C, Harel N, Howell M J, MacKinnon C D, Vitek J L, Johnson L A. Basal ganglia engagement during REM sleep movements in Parkinson's disease[J]. NPJ Parkinson's disease, 2022, 8(1): 116.

Data exclusions	Five subjects (PD-2/10 and Dyst-10/11/13) were excluded from the REM sleep analysis as they had low accurately diagnosed REM sleep segment count (n < 5).
Replication	The main findings that pallidal beta power was exaggerated in PD patients were successfully replicated (a) in the beta burst analysis and (b) after spectral parameterizations. Since this is the first study comparing beta power during sleep between PD and dystonia patients, to the best of our knowledge we do not know other independent groups that have replicated our findings, but we expect it will be replicated in the near future.
Randomization	This is not relevant to our study as all included subjects are in the experimental group.
Blinding	Since this study is not related to interventions and therapeutic effects (i.e., we recorded brain signals from two groups of patients during sleep), blinding was not relevant to the study.

Reporting for specific materials, systems and methods

We require information from authors about some types of materials, experimental systems and methods used in many studies. Here, indicate whether each material, system or method listed is relevant to your study. If you are not sure if a list item applies to your research, read the appropriate section before selecting a response.

Materials & experimental systems

n/a	Involved in the study
☒	☐ Antibodies
☒	☐ Eukaryotic cell lines
☒	☐ Palaeontology and archaeology
☒	☐ Animals and other organisms
☒	☐ Clinical data
☒	☐ Dual use research of concern

Methods

n/a	Involved in the study
☒	☐ ChIP-seq
☒	☐ Flow cytometry
☐	☒ MRI-based neuroimaging

Magnetic resonance imaging

Experimental design

Design type	Not applicable. We only used structure MRI (T1 sequence) for DBS electrode reconstruction
Design specifications	Not applicable
Behavioral performance measures	Not applicable

Acquisition

Imaging type(s)	Structure MRI
Field strength	3.0 T
Sequence & imaging parameters	MRIs were acquired for all participants using a 3T Siemens Verio scanner with a T1-MPRAGE sequence [repetition time (TR) =2,300 ms, echo time (TE) =2.53 ms, flip angle =12°, slice thickness =1 mm, no gap, voxel size =1 mm × 1 mm × 1 mm]
Area of acquisition	Whole brain scan

Diffusion MRI ☐ Used ☒ Not used

Preprocessing

Preprocessing software	We used the advanced electrode localization pipeline (in Lead-DBS version 2.5.3) with default settings for electrode reconstruction and group analysis in Montreal Neurological Institute (MNI) space
Normalization	The pre-operative MRI scan (T1 sequence) was linearly co-registered with the postoperative anatomical CT and nonlinearly warped to the MNI template (ICBM 2009bNonlinear Asymmetric) using the Advanced Normalization Tools (ANTs)
Normalization template	ICBM 2009bNonlinear Asymmetric
Noise and artifact removal	Visual identification

Volume censoring	Not applicable

Statistical modeling & inference

Model type and settings	Not applicable
Effect(s) tested	Not applicable

Specify type of analysis: ☐ Whole brain ☐ ROI-based ☐ Both

Statistic type for inference (See Eklund et al. 2016)	Not applicable
Correction	Not applicable

Models & analysis

n/a	Involved in the study
☒	☐ Functional and/or effective connectivity
☒	☐ Graph analysis
☒	☐ Multivariate modeling or predictive analysis
